# LH supplementation in ovarian stimulation: propensity score and generalized estimating equations analysis over 2000 embryos

**DOI:** 10.3389/fendo.2026.1846779

**Published:** 2026-05-22

**Authors:** Merve Dizdar, Fazilet Kübra Boynukalın, Betül Dündar, Meral Gültomruk, İbrahim Kale, Zalihe Yarkıner, Mustafa Bahçeci, Nikolaos P. Polyzos, Gürkan Bozdağ

**Affiliations:** 1 Department of Obstetrics and Gynecology, Umraniye Training and Research Hospital, İstanbul, Türkiye; 2 Department of Clinical Trials, London School of Hygiene and Tropical Medicine, London, United Kingdom; 3 Department of Infertility, Bahceci Fulya IVF Center, Istanbul, Türkiye; 4 Department of Infertility, Bahceci Bursa IVF Center, Bursa, Türkiye; 5 Department of Research and Development, Bahceci Fulya IVF Center, Istanbul, Türkiye; 6 Department of Basic Sciences and Humanities, Cyprus International University, Nicosia, Cyprus; 7 Department of Reproductive Medicine, Dexeus University Hospital, Barcelona, Spain

**Keywords:** embryo euploidy, frozen embryo transfer (FET), luteinizing hormone, ovarian stimulation, PGT-A

## Abstract

**Research question:**

Does luteinizing hormone (LH)-activity supplementation during ovarian stimulation (OS) influence embryo-ploidy rate compared with follicle-stimulating hormone (FSH)-only stimulation in *in vitro* fertilization (IVF) cycles with preimplantation genetic testing for aneuploidy (PGT-A)?

**Methods:**

Retrospective single-center cohort study (January 2018–January 2024) including 4,417 IVF/PGT-A cycles using gonadotropin-releasing hormone antagonist or progesterone-primed protocols. After exclusions, 952 cycles were selected via 1:1 propensity score-matching for female age and oocyte yield: 476 OS with recombinant FSH alone and 476 with added LH activity (recombinant LH or human menopausal gonadotropin). As the primary outcome, euploidy was assessed by trophectoderm biopsy and next-generation sequencing. Generalized estimating equations accounted for within-patient clustering. Secondary endpoints were clinical pregnancy, live birth, and miscarriage after frozen euploid embryo transfer (FEET).

**Results:**

There were no significant differences between FSH-only and FSH + LH activity groups with regard to oocyte yield, maturation, fertilization rates, or embryo euploidy rates (45.8% vs. 45.2%; p=0.749). Female age was identified as an independent negative predictor of euploidy (OR = 0.899; p< 0.001), while good-quality embryos had significantly higher odds of being euploid compared with poor-quality embryos (OR = 2.053; p< 0.001). Secondary outcomes showed no significant differences in clinical pregnancy rate, live birth rate, or miscarriage rate following FEET between groups. The addition of LH activity during ovarian stimulation was not independently associated with embryo ploidy status or live birth outcomes.

**Conclusions:**

In a large matched IVF/PGT-A cohort, LH activity supplementation during OS did not improve blastocyst euploidy or reproductive outcomes. Gonadotropin regimens should be individualized rather than routinely including LH in unselected patients.

## Introduction

Luteinizing hormone (LH) is a key endocrine regulator of the natural ovulatory cycle, with a central role in follicular maturation and the induction of ovulation (1). In *in vitro* fertilization (IVF), ovarian stimulation (OS) is typically achieved using exogenous gonadotropins to promote the development of multiple follicles. While follicle-stimulating hormone (FSH) is universally recognized as essential for effective OS, the role and necessity of exogenous LH supplementation remains unclear.

Endogenous LH concentrations vary considerably among individuals, particularly in women of advanced reproductive age, low ovarian responders, and patients undergoing gonadotropin-releasing hormone (GnRH) agonist or antagonist protocols, which markedly suppress LH secretion (2, 3). Although previous studies have shown that serum LH levels decrease with advancing age (4), evidence remains inconclusive regarding which subgroups of patients may actually benefit from LH supplementation. Nevertheless, apart from patients with hypogonadotropic hypogonadism, LH has been frequently considered for the treatment of advanced age women (36–40 years) and those with poor ovarian response (5).

Proposed benefits of LH effect on these patients include enhanced follicular responsiveness, improved oocyte competence, and increased pregnancy rates in selected patients. However, outcomes in unselected IVF populations are inconsistent, possibly due to LH-induced atresia of small antral follicles that lack LH receptors and depend solely on FSH (6).

Although the effect of LH on oocyte meiosis and chromosomal integrity remains unclear, early studies supported that excessive or premature LH exposure may disrupt meiotic timing and spindle formation, potentially increasing aneuploidy risk (7). More recently others evaluated the relationship between gonadotropin type and embryo ploidy however, both studies were limited by small sample sizes and narrow inclusion criteria (8, 9). The most recent report was a small study concluded that stimulation with r-FSH/r-LH was associated with lower aneuploidy rate and significantly higher live birth rates (10).

Given the conflicting evidence, we conducted a study aiming to investigate whether LH supplementation during OS may be associated with embryo euploidy rates as compared with FSH-only stimulation in a large real-world IVF cohort, and to assess subsequent reproductive outcomes following frozen euploid embryo transfer (FEET) cycles.

## Materials and methods

### Study population and design

This retrospective cohort study was conducted at a single tertiary *in vitro* fertilization (IVF) center between January 2018 and January 2024. All data were extracted from the center’s electronic medical record system. Eligible participants were women aged 18–45 years undergoing preimplantation genetic testing for aneuploidy (PGT-A) for indications including advanced maternal age, recurrent pregnancy loss, and recurrent implantation failure.

A total of 19,237 IVF cycles were reviewed. Inclusion criteria were cycles using gonadotropin-releasing hormone (GnRH) antagonist or progesterone-primed ovarian stimulation (PPOS) protocols. Exclusion criteria included GnRH agonist protocols, random-start stimulation, and PGT for monogenic diseases (PGT-M) or structural rearrangements (PGT-SR). After exclusions, 4,417 cycles were eligible. Propensity score matching (based on female age and oocyte yield) produced 952 matched cycles: 476 stimulated with recombinant FSH alone and 476 with added LH activity (recombinant LH or human menopausal gonadotropin).

### Ovarian stimulation protocols

Ovarian stimulation commenced in the early follicular phase with either recombinant FSH (Gonal-F^®^, Merck Serono; or Puregon^®^, Organon) alone or in combination with recombinant LH (Pergoveris^®^, Merck Serono) or hMG-based LH activity (Menopur^®^, Ferring; Meriofert^®^, IBSA). The choice and initial dosage were based on age, ovarian reserve, and clinician judgment.

PPOS cycles received dydrogesterone (Duphaston^®^, Abbott) or medroxyprogesterone acetate (Tarlusal^®^, Deva) orally. In GnRH antagonist cycles, cetrorelix acetate (Cetrotide^®^, Merck Serono; or Oxotide^®^, VEM) was initiated per fixed or flexible protocol. Final oocyte maturation was triggered with choriogonadotropin alfa (Ovitrelle^®^, Merck Serono) and/or triptorelin acetate (Gonapeptyl^®^, Ferring) when the lead follicle was ≥17 mm. Oocyte retrieval occurred 35–36 hours post-trigger.

### Laboratory procedures

Oocyte retrieval, denudation, intracytoplasmic sperm injection, embryo culture, vitrification, and warming followed standard protocols (11). Blastocyst morphology was graded per Gardner and Schoolcraft criteria (12). Assisted hatching was performed on day 3. Trophectoderm biopsy was conducted using the pulling method; 5–8 cells were collected and immediately placed into PCR tubes.

### Preimplantation genetic testing

Whole-genome amplification, DNA barcoding, and 24-chromosome aneuploidy screening were performed using the Ion ReproSeq™ PGS Kit, automated on the IonChef™ and sequenced on the Ion S5 System. Data were analyzed with Ion Reporter software (hg19 reference). Embryos were classified as euploid (no aneuploidy or <50% mosaicism) or aneuploid.

### Endometrial preparation and frozen embryo transfer

Frozen euploid embryo transfer (FEET) cycles used hormone replacement therapy, modified natural cycle, or mild ovarian stimulation protocols. Protocol-specific monitoring, hormone administration, ovulation triggering, and luteal support followed standard clinical practice, with embryo transfer performed under transabdominal ultrasound guidance.

### Outcome measures

The primary outcome was euploidy rate per biopsied embryo. Secondary outcomes included euploidy per metaphase II oocyte and per normally fertilized oocyte (2PN), clinical pregnancy rate (CPR), and live birth rate (LBR) per euploid embryo transfer. CPR was defined as an intrauterine gestational sac on ultrasound; LBR as delivery beyond 24 weeks’ gestation.

### Statistical analysis

Continuous variables are reported as mean ± standard deviation; categorical variables as number and percentage. Group comparisons used the Mann–Whitney U test for non-normal data and Chi-square test for categorical data. Propensity score matching (PSM) was performed using 1:1 nearest-neighbor matching (covariates: age, number of oocytes retrieved). The effect of LH supplementation on euploidy was evaluated using generalized estimating equations (GEE) with binomial distribution and logit link, accounting for embryo clustering. Odds ratios (OR) and 95% confidence intervals (CI) were reported. Analyses were conducted using SPSS version 26.0 and R version 4.0.2.

## Results

### Basal characteristics

**Figure 1:**
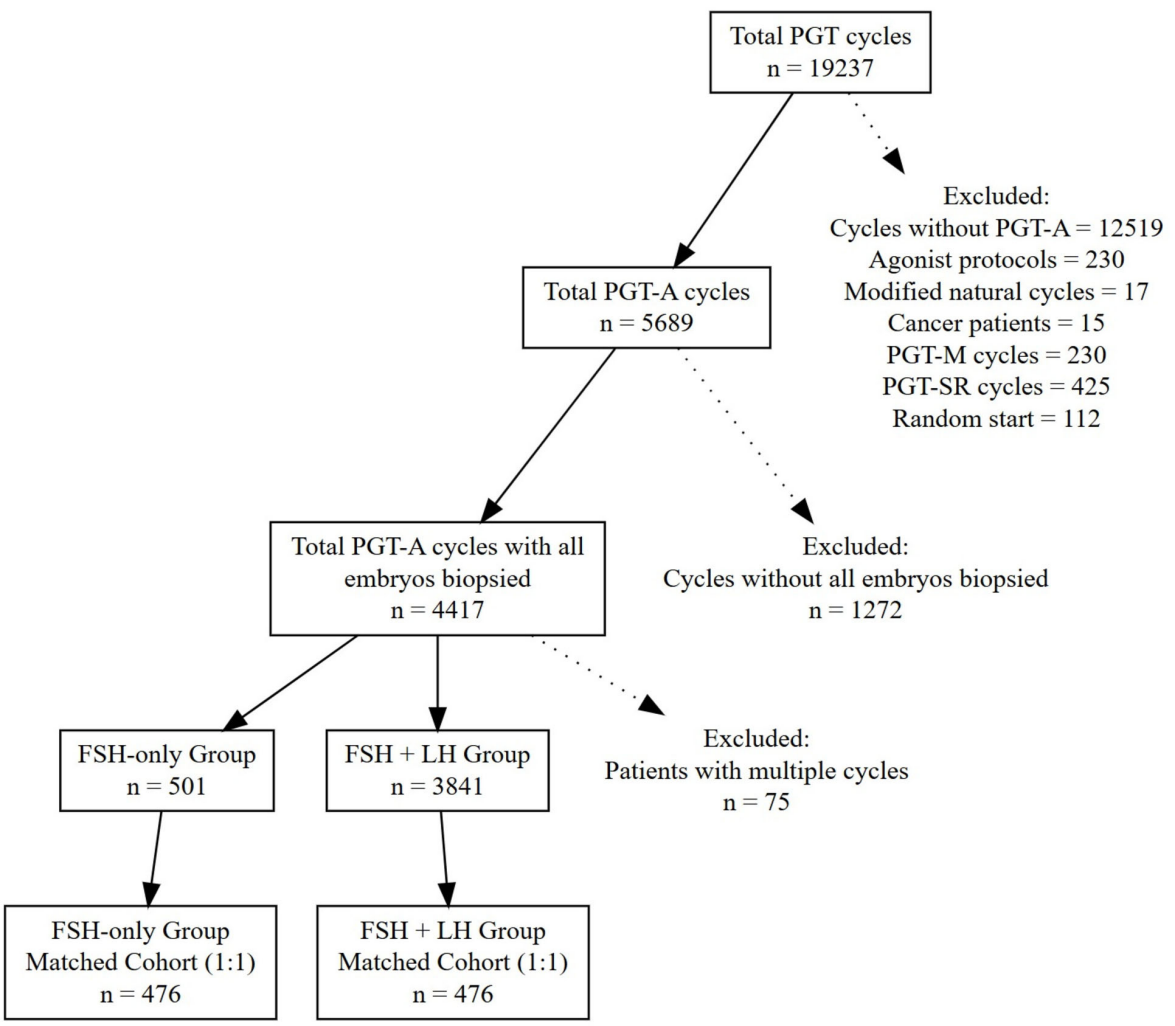
Flowchart of patient selection for the study.

A total of 5,689 PGT-A cycles were analyzed, including 4,801 with FSH + LH activity and 888 with FSH only. Among the FSH-only group, 501 patients had all embryos biopsied and among FSH + LH activity group 3841 patients had all embryos biopsied. Due to baseline differences between groups, PSM was performed based on female age and oocyte count. After matching, 476 patients remained in each group (**Figure 1**). No significant differences were observed between the groups in terms of female age (34.85 ± 5.8 vs. 34.96 ± 6.4 years, p=0.786), male age (36.88 ± 6.7 vs. 37.38 ± 7.1 years, p = 0.259), or duration of stimulation (11.58 ± 1.7 vs. 11.46 ± 1.6 days, p=0.279). Although body mass index (BMI) was significantly lower in patients receiving LH activity supplementation (23.8 ± 4.1 vs. 25. 5 ± 4.9 kg/m², p< 0.01), there were no significant differences in the total gonadotropin dose administered or in serum E2 and P levels on trigger day. As shown in **Table 1**, oocyte retrieval outcomes; the number of COCs and metaphase II (MII) oocytes were comparable between groups, as were oocyte maturation and fertilization rates (p> 0.05 for all comparisons).

**Table 1:** Baseline characteristics of patients before and after propensity score matching.

	Before propensity score matching			After propensity score matching		
	FSH-only n= 501	FSH + LH activity n=3686	p-value	FSH-only n= 476	FSH + LH activity n= 476	p-value
Female age (y)	34.85 ± 5.8	37.72 ± 4.9	<0.001	34.85 ± 5.8	34.96 ± 6.4	0.786
Male age (y)	36.99 ± 6.6	39.71 ± 6.6	<0.001	36.88 ± 6.7	37.38 ± 7.1	0.259
BMI (kg/m2)	22.9 ± 9.1	21.9± 9.1	0.018	25.5 ± 4.9	23.8± 4.1	<0.01
Duration of stimulation	11.58 ± 1.8	11.55 ± 1.7	0.651	11.58 ± 1.7	11.46 ± 1.6	0.279
OS protocol			<0.001			<0.001
GnRH Antagonist	365/501(72.9)	3030/3686(82.1)		344/476(72.3)	387/476(81.3)	
PPOS	136/501(27.1)	656/3686(18.9)		132/476(27.7)	89/476(18.7)	
Total GN dosage (IU)	2300 ± 946	2651 ± 1251	<0.001	2277 ± 755	2205 ± 720	0.137
Trigger day E2 (pg/ml)	2055 ± 2053	1415 ± 1466	<0.001	2010 ± 1877	1966 ± 1970	0.726
Trigger day P4 (ng/dl)	0.7 ± 0.8	0.6 ± 0.7	<0.001	0.7 ± 0.7	0.7 ± 0.6	0.3
No of COC	13.3 ± 8.9	8.92 ± 5.98	<0.001	12.9 ± 7.6	12.4 ± 7.7	0.411
No of MII	10.3 ± 7.3	6.9 ± 4.9	<0.001	9.9 ± 6.3	9.5 ± 6.4	0.732
No of 2PN	7.7 ± 5.4	5.4 ± 3.8	<0.001	7.5 ± 4.8	7.4 ± 5.3	0.165
Maturation rate (%)	78.9 ± 17.5	78.9 ± 18.8	0.926	78.4 ± 17.4	77.6 ± 18.2	0.51
Fertilization rate (%)	77.9 ± 18.4	81 ± 19.1	<0.001	78.8 ± 18	79.6 ± 18.6	0.497
Blastocyst development rate (%)	39 ± 24.8	48.4 ± 27.1	<0.001	38.9 ± 23.7	41.5 ± 24.7	0.093
Male factor			0,228			0,335
Absence (%)	436/501 (87)	3128/3686 (84.9)		419/476 (88)	409/476 (85.9)	
Presence (severe) (%)	65/501 (13)	558/3686 (15.1)		57/476 (12)	67/476 (14.1)	

Values are expressed as mean ± SD, unless stated otherwise. E2, Estradiol; P4, Progesterone; COC, Cumulus oocyte complexes; MII, Metaphase-II oocyte. Maturation rate was calculated as the number of metaphase II (MII) oocytes divided by the total number of collected cumulus–oocyte complexes (COCs). The blastocyst development rate was defined as the number of blastocysts formed on Day 5 or 6 divided by the total number of 2PN embryos.

### Outcomes

No statistically significant differences were observed in embryo euploidy outcomes between patients who received FSH-only stimulation and those who received FSH combined with LH activity (**Table 2**). The mean number of embryos biopsied per patient was comparable between the two groups (2.41 ± 1.6 vs. 2.34 ± 1.5; p=0.506), as was the mean number of euploid blastocysts (1.09 ± 1.2 vs. 1.0 ± 1.1; p=0.845). The primary outcome of the study, the euploidy rate per biopsied embryo was 45.8% in the FSH-only group and 45.2% in the group receiving FSH with LH supplementation (p=0.749). When calculated based on the number of COCs and MII, the respective euploidy rates were 10.8% and 11.4% (p=0.359). Similarly, the euploidy rate per 2PN embryo did not differ significantly between the groups (14.2% vs. 14.7%; p=0.597).

**Table 2:** Comparison of embryo euploidy rates between FSH-only and LH-activity stimulation groups.

	Before propensity score matching			After propensity score matching		
	FSH-only n= 501	FSH + LH activity n=3686	p-value	FSH-only n= 476	FSH + LH activity n= 476	p-value
No of embryos biopsied	2.39 ± 1.59	2.05 ± 1.3	<0.001	2.34 ± 1.5	2.41 ± 1.6	0.506
No of euploid blastocysts	1.09 ± 1.1	0.74 ± 0.9	<0.001	1.07 ± 1.1	1.09 ± 1.2	0.845
Total Embryos						
Euploidy rate per M-II (%)	545/7018 (7.8)	2737/25500(10.7)	<0.001	510/4715 (10.8)	517/4529 (11.4)	0.359
Euploidy rate per 2PN (%)	545/5414 (10.1)	2737/20019(13.7)	<0.001	510/3588 (14.2)	517/3528 (14.7)	0.597
Euploidy rate per biopsied embryo (%)	545/1195 (45.6)	2737/7545 (36.3)	<0.001	510/1113 (45.8)	517/1145 (45.2)	0.749
Per patient analysis						
Euploidy rate /per M2	12.6 ± 15.9	12.9 ± 18.9	0.682	13 ± 16.2	14.1 ± 18.1	0.290
Euploidy rate /per 2PN	16.1 ± 18.8	16.2 ± 23.1	0.91	16.6 ± 19.1	18 ± 22	0.279
Euploidy rate /per embryo	44.1 ± 39	34.6 ± 39.8	<0.001	44.1 ± 39	43.8 ± 39.4	0.898
Probability of having at least one euploid embryo (%)	323/501(64.5)	1853/3686(50.3)	0.001	307/476 (64.5)	306/476 (64.3)	0.946

Values are expressed as mean ± SD, unless stated otherwise. Euploidy rate was defined as the number of euploid embryos divided by the number of M-II oocytes collected, 2PN or embryos biopsied.

In the per-patient analysis, the mean euploidy rate per embryo was 44.1 ± 39.0% in the FSH-only group and 43.8 ± 39.4% in the FSH + LH activity group (p=0.898). Likewise, the mean euploidy rates per MII oocyte (13.0 ± 16.2% vs. 14.1 ± 18.1%; p=0.290) and per 2PN embryo (16.6 ± 19.1% vs. 18.0 ± 22.0%; p=0.279) were also statistically similar. Importantly, the probability of obtaining at least one euploid embryo per cycle was not significantly different between the two groups (64.5% vs. 64.3%; p=0.946).

A GEE model was employed to identify independent predictors of embryo biopsy outcome, defined as euploid versus aneuploid. The analysis included 2258 embryos, of which 45.5% were classified as euploid and 54.5% as aneuploid (**Table 3**). As expected, female age emerged as a significant predictor, with increasing age being inversely associated with the likelihood of embryo euploidy (OR:0.899; 95%CI:0.882–0.915; p < 0.001), indicating a consistent and statistically robust negative association. Embryo morphological quality was also independently associated with biopsy outcome. Embryos graded as good demonstrated significantly higher odds of being euploid compared to those graded as poor (OR:2.053; 95%CI:1.414–2.981; p < 0.001), while those with moderate-quality scores did not significantly differ from the poor-quality group. In contrast, other variables including the use of LH activity during ovarian stimulation, duration of stimulation, total FSH dose, total gonadotropin dose, the type of ovulation trigger, presence of male factor infertility (defined by abnormal semen parameters per WHO criteria), and the day of trophectoderm biopsy (Day 5 vs. Day 6) were not significantly associated with embryo ploidy status (p > 0.05 for all; **Table 3**).

**Table 3:** Generalized estimating equations (GEE) model for predictors of euploid biopsy results.

Parameter	OR	95% CI (Lower-Upper)	p-value
Male Factor			
Present vs. Absent	1.220	(0.938-1.587)	0.138
LH-activity			
Absent vs. Present	1.004	(0.830-1.213)	0.970
Day of biopsy			
Day 5 vs. Day 6	1.416	(0.693-2.893)	0.340
Quality of blastocyst			
Good vs. Poor	2.053	(1.414-2.981)	< 0.001
Moderate vs. Poor	1.283	(0.900-1.829)	0.168
Type of trigger			
hCG vs. Dual	0.962	(0.734-1.261)	0.778
GnRH agonist vs. Dual	0.997	(0.795-1.250)	0.980
Protocol of OS			
GnRHant vs. PPOS	0.855	(0.688-1.063)	0.159
Female Age	0.899	(0.882-0.915)	<0.001
Total FSH Dose	1.000	(1.000-1.000)	0.244
Duration of OS	1.053	(0.974-1.137)	0.194

Dual trigger represents concomitant utilization of hCG and GnRH agonist 35–36 hours before oocyte collection. OS, Ovarian stimulation.

The respective number of FEET cycles were 283 and 277 from cycles that had been stimulated with FSH-only and FSH + LH activity group. No statistically significant differences were observed between the two groups in terms of CPR, LBR, or miscarriage rate. The CPR was 60.4% (171/283) in the FSH-only group and 64.6% (179/277) in the FSH + LH activity group (**Table 4**, *p* = 0.337). Similarly, the LBRs were 50.5% (143/283) and 52.3% (145/277) in the respective groups (*p* = 0.673). The miscarriage rates among patients with a confirmed clinical pregnancy was also comparable: 14.0% (24/171) in the FSH-only group versus 12.3% (22/179) in the LH-supplemented group (*p* = 0.658).

**Table 4:** Comparison of demographic features and embryological data of patients with frozen euploid embryo transfer (FEET) cycles after an ovarian stimulation with FSH-only or with some LH-activity.

	FSH-only n= 283	FSH + LH activity n= 277	p-value
Female age (y)	32.8 ± 4.8	32.8 ± 5	0.973
Male age (y)	35.3 ± 6.2	35.8 ± 5.6	0.301
BMI (kg/m2)	35.3 ± 4.9	23.8 ± 4.2	0.01
Quality of blastocyst (%)			0.682
Good	116/303 (38.3)	101/290 (34.8	
Moderate	167/303 (55.1)	169/290 (58.3)	
Poor	20/303 (6.6)	20/290 (6.9)	
No of embryos transferred (%)			0.282
1	263/283 (92.9)	264/277 (95.3)	
2	20/263 (7.1)	13/277 (4.7)	
Day of blastocyst development (%)			0.12
5	169/303 (55.8)	180/290 (62.0)	
6	134/303 (44.2)	110/290 (38.0)	
Endometrial thickness (mm)	8.7 ± 6	9.4 ± 5	0.365
Distribution of FEET protocol (%)			0.055
HRT	236/283 (83.4)	247/277 (89.2)	
Natural cycle (%)	16/283 (5.6)	15 /277 (5.4)	
Mild-Stimulation (%)	31/283 (11)	15 /277 (5.4)	
CPR (%)	171/283 (60.4)	179/277 (64.6)	0.337
LBR (%)	143/283 (50.5)	145/277 (52.3)	0.673
Miscarriage rate (%)	24/171 (14)	22/179 (12.3)	0.658
Ectopic pregnancy (%)	4/171 (2.3)	0	

Values are expressed as mean ± SD, unless stated otherwise. HRT, Hormone-replacement; CPR, Clinical pregnancy rate; LBR, Live birth rate.

In the regression analysis evaluating factors associated with live birth following FEET, none ofthe assessed variables, including the presence of LH activity during OS were found to be statistically significant predictors other than transfer day (Day 5 vs. Day 6) (**Supplementary Table 1**).

## Discussion

This study comprehensively evaluated the impact of LH supplementation to FSH during OS on two critical reproductive outcomes: embryo euploidy rates and pregnancy outcome parameters following a FEET. Despite theoretical and physiological rationale supporting a potential role for LH activity in folliculogenesis and oocyte maturation, our results demonstrate that the addition of LH did not significantly influence the rate of ploidy status, nor did it confer any measurable improvement in pregnancy outcomes in the context of FEET cycles.

Although clinical data remain inconclusive, mechanistic insights from *in vitro* studies offer valuable perspectives on the potential impact of LH activity on folliculogenesis and oocyte competence. The LH plays a pivotal role in promoting and accelerating follicular development across multiple stages of folliculogenesis (13). Specifically, LH stimulates androgen production by theca cells, which in turn supports estrogen synthesis and promotes follicular growth (14). While LH supplementation may enhance oocyte quality through various mechanisms, its effects are not universally beneficial. Early studies suggested that excessive LH may prematurely trigger meiosis by inhibiting oocyte maturation inhibitor, leading to post-mature oocytes with lower developmental potential (7). Later findings support that LH indirectly induces meiosis, promotes steroidogenesis, and reduces granulosa cell proliferation (15, 16). These findings raise concerns that premature or dysregulated LH signaling may impair oocyte quality and embryonic potential in certain clinical contexts. This view is further supported by the work of Filicori et al. and showed that exogenous LH selectively promotes dominant follicle growth while inducing atresia in smaller ones, influencing folliculogenesis by stage (17). However, its potential impact on oocyte chromosomal integrity and euploidy remains unclear. In addition, recent evidence from capacitation invitro maturation studies highlights the importance of transzonal projections in maintaining oocyte–cumulus communication and regulating meiotic arrest (18). The physiological LH surge induces an ovulatory cascade leading to transzonal projections withdrawal and meiotic resumption, and premature activation of this pathway in small or immature follicles has been proposed to impair oocyte competence. However, these observations largely derive from *in vitro* or non-physiological settings. In our study, LH exposure remained within physiological ranges, and no differences were observed in oocyte yield or embryo euploidy, suggesting that such mechanisms may not translate into measurable clinical effects in conventional IVF practice.

The impact of gonadotropin type on embryo euploidy remains a subject of ongoing debate, with existing studies yielding inconsistent and often inconclusive findings. Cascales et al. evaluated various stimulation parameters, including gonadotropin type, and found no significant association with embryo aneuploidy or mosaicism rates (19). However, the sample sizes per gonadotropin subgroup were limited and only two patients received gonadotropins with LH activity, thus significantly reducing the statistical power to detect meaningful differences for a LH activity. Similarly, Vaiarelli et al., in a matched case–control study involving women of advanced maternal age, reported no association between gonadotropin regimen and euploid blastocyst rate (8). They suggested that when OS is appropriately individualized, oocyte competence may be largely independent of gonadotropin type. Yet, the relatively small subgroup sizes in that study, ranging from 55 to 127 cycles per arm, again raise concerns regarding statistical robustness. In contrast, McCulloh et al. found a significant association between hMG use and increased euploidy rates in donor oocyte cycles. The study retrospectively analyzed 226 donor IVF cycles, comparing outcomes between those stimulated with recombinant FSH alone vs. hMG-based protocols (20). The authors observed that embryos derived from oocytes exposed to hMG had a significantly higher rate of euploidy, as assessed by array comparative genomic hybridization (aCGH), suggesting a potential beneficial role of LH activity in promoting chromosomal competence. The authors proposed that the presence of LH activity may enhance oocyte developmental potential by improving steroidogenesis, supporting cytoplasmic maturation, and stabilizing meiotic spindle formation, ultimately leading to improved chromosomal segregation. The study minimized confounding by using oocytes from young, healthy donors and applying uniform lab protocols at a single center, enhancing internal validity. However, limitations include its retrospective design, the use of less sensitive aCGH instead of NGS, and limited generalizability to older or subfertile patients in autologous cycles. A similar findings were reported in a retrospective, paired cohort, single-center study of whom 141 were stimulated with recombinant FSH plus recombinant LH (r-FSH/r-LH) and 146 received highly purified human menopausal gonadotropin (HP-HMG) (10). The authors reported a lower aneuploidy rate, while a significantly higher live birth and clinical pregnancy rates in embryos from women stimulated with r-FSH/r-LH than HP-HMG in that poster presentation.

Compared to previous studies (8, 19, 20) a key strength of the present investigation lies in its methodological rigor. Most notably, the use of a GEE model to account for the clustering of embryos within individual patients, an approach not employed in earlier work. The study also benefits from a substantially larger and statistically robust cohort, comprising 952 patients and over 2,000 embryos, which provides a solid foundation for assessing the relationship between gonadotropin composition and embryo euploidy. These design features improve both the precision of the analysis and the broader applicability of the findings. In contrast to earlier studies constrained by small sample sizes and limited adjustment for confounding variables, our objective was to build upon and validate these prior observations within a larger, well-controlled cohort.

Embryo chromosomal competence is widely understood to be primarily determined by patient-specific factors; most notably, female age rather than by the specific ovarian stimulation protocol employed (19). Multiple studies have demonstrated that variations in stimulation strategy do not significantly impact euploidy rates as determined by PGT-A. For instance, comparable euploidy outcomes have been reported between PPOS and GnRH antagonist protocols, suggesting that the type of regimen may have limited influence on chromosomal integrity (21). Additionally, prior research indicates that neither the total gonadotropin dosage nor the magnitude of ovarian response are major determinants of embryo euploidy (22–24).

However, a critical and often overlooked question remains: even when an embryo is confirmed to be euploid, could the type of gonadotropin or stimulation protocol used during its development still influence its implantation potential or overall developmental competence? This consideration is particularly relevant in the context of FEET cycles, where chromosomal quality has already been accounted for, yet subtle biological effects of the stimulation regimen may persist and influence downstream outcomes. In this regard, the findings of our study suggest that embryos derived from stimulation protocols with or without LH activity exhibit comparable implantation potential, thereby indicating that their developmental competence is not significantly affected by the type of LH activity used during OS.

This study has several limitations that should be acknowledged. First, although PSM was employed to minimize selection bias, the retrospective design inherently limits the ability to establish causality. Second, the LH-containing group included both recombinant LH and hMG which may introduce biological heterogeneity despite their comparable LH bioactivity. Third, mosaicism status was not assessed in the genetic analysis, potentially limiting the interpretation of embryo competence and chromosomal integrity. Mosaicism below 30% could not be reliably detected, while embryos with mosaicism levels above 50% were classified as aneuploid. Therefore, the analyzed cohort represents an intermediate group, excluding both very low- and high-level mosaic cases. Although this may limit direct comparability with studies using different thresholds, the consistent application of these criteria across all samples ensures a uniform and internally coherent dataset. Importantly, this did not translate into any observable difference in reproductive outcomes, suggesting that the impact of this limitation on our conclusions is likely minimal. Despite these limitations, the study presents several important strengths. Foremost among them is the large and adequately powered cohort, consisting of nearly 1,000 patients and over 2,000 embryos overall even after following PSM. This extensive sample size provides a solid basis for detecting clinically meaningful differences and enhances the statistical robustness of the findings. Additionally, the use of GEE enabled appropriate adjustment for the clustering of embryos within individual patients, further strengthening the analytical validity. The application of NGS for preimplantation genetic testing ensured high-resolution chromosomal evaluation, while restricting clinical outcome analysis to euploid ETs minimized confounding from embryonic aneuploidy. Collectively, these methodological strengths significantly improve the internal validity and clinical applicability of our results.

In conclusion, this study provides comprehensive evidence that LH supplementation during OS is not associated with embryo euploidy rates or clinical outcomes following frozen euploid ET. Despite biologically plausible mechanisms and supportive *in vitro* findings suggesting a potential role for LH in oocyte maturation and chromosomal integrity, embryos derived from protocols with or without LH activity demonstrated comparable developmental competence and implantation potential. By analyzing a large PSM cohort with robust statistical modeling and high-resolution genetic screening via NGS, this investigation overcomes key limitations of earlier studies and reinforces the importance of individualized treatment strategies.

## Data Availability

Data is available at OSF Depository: https://osf.io/rs2au.

## Supplementary material

Supplementary Table 1 Regression analysis for factors affecting live birth. OR: Odds Ratio CI: Confidence Interval.
