# Supplementary material for: LH supplementation in ovarian stimulation: propensity score and generalized estimating equations analysis over 2000 embryos
Source: Front Endocrinol (Lausanne). 2026 May 22;17:1846779. doi: 10.3389/fendo.2026.1846779 (PMC13236556; doi:10.3389/fendo.2026.1846779)
Supplement: Supplementary Table 1 — Regression analysis for factors affecting live birth. OR: Odds Ratio CI: Confidence Interval. [file Table1.pdf]

**Supplementary Table 1:** Regression analysis for factors affecting live birth

| Variable | OR | 95% CI | p-value |
|---|---|---|---|
| Female age (years) | 1.002 | 0.968 - 1.038 | 0.903 |
| BMI ($kg/m^2$) | 0.977 | 0.941 - 1.014 | 0.215 |
| Presence of LH activity | 0.994 | 0.704 - 1.403 | 0.973 |
| No. of embryos transferred | 1.031 | 0.501 - 2.123 | 0.935 |
| Embryo day (ref: Day 5) | | | <0.001 |
| Day 6 | 0.519 | 0.363 - 0.742 | |
| Embryo quality (ref: good quality) | | | |
| Moderate | 0.882 | 0.615 - 1.265 | 0.494 |
| Poor | 0.780 | 0.353 - 1.727 | 0.541 |
| Endometrial thickness (mm) | 0.996 | 0.965 - 1.029 | 0.826 |

OR: Odds Ratio CI: Confidence Interval
